# A descriptive study of Forcefully Displaced Myanmar Nationals (FDMN) presenting for care at public health sector hospitals in Bangladesh

**DOI:** 10.1080/16549716.2021.1968124

**Published:** 2021-09-08

**Authors:** Rumana Rashid, Abu Muhammad Shamsu Uddin, Pu Chaw Nu, Abdus Salam, Sumon Barua, Abdul Mannan, Mohammed Shahjahan, Misbah Uddin Ahmed, Ridwanur Rahman, Arjen Dondorp, Richard J Maude, Jaqueline Deen, Lorenz von Seidlein, Mohammad Abul Faiz

**Affiliations:** a Epidemiology and Community Medicine, Bangladesh Institute of Tropical and Infectious Diseases (BITID) Faujdarhat, Chattogram, Bangladesh; b Internal Medicine, Sadar Hospital, Cox’s Bazar, Bangladesh; c Civil Surgeon (Former), Cox’s Bazar, Bangladesh; d Upazila Health and Family Planning Officer (UHFPO), Teknaf, Cox’s Bazar, Bangladesh; e Upazila Health and Family Planning Officer (UHFPO), Ukhiya, Cox’s Bazar, Bangladesh; f Internal Medicine, Cox’s Bazar Medical College, Cox’s Bazar, Bangladesh; g Assistant Director, National Institute of Kidney Diseases, Dhaka, Bangladesh; h Research Centre, Universal Medical College, Dhaka, Bangladesh; i Mahidol Oxford Tropical Medicine Research Unit, Faculty of Tropical Medicine, Mahidol University, Bangkok, Thailand; j Centre for Tropical Medicine and Global Health, Nuffield Department of Medicine, University of Oxford, Oxford, UK; k Harvard T.H. Chan School of Public Health, Harvard University, Boston, USA; l The Open University, Milton Keynes, UK; m Child Health, Institute of Child Health and Human Development, National Institutes of Health, University of the Philippines, Manila, Philippines; n Director General of Health Services, Dhaka, Bangladesh (Retired); o Dev Care Foundation, Dhaka, Bangladesh

**Keywords:** Myanmar, refugees, Rohingya, Health services, infectious diseases, pregnancy, trauma

## Abstract

**Background:**

In 2017 hundreds of thousands of ‘Rohingya’ fled to camps for Forcefully Displaced Myanmar Nationals (FDMN) in Cox’s Bazar, Bangladesh.

**Objective:**

To describe the FDMNs presenting for care at public health facilities in Bangladesh so as to understand the health problems faced by the FDMNs and the burden on these public health facilities.

**Methods:**

This study combined a retrospective review of existing hospital and clinic data with prospective surveillance in government health care centres.

**Findings:**

The retrospective data showed a 26% increase in the number of consultations at the Kutupalong community clinic, the primary health facility closest to the camps, from 19,567 in 2015 to 26,309 in 2019. There was a corresponding 11% increase in admissions to health facilities in the area, from 80,991 in 2017 to 91,424 in 2019. Prospective surveillance of 9,421 FDMNs seeking health care from July 2018 to December 2019 showed that 29% had an infectious disease, 20% nutritional problems, 12% pregnancy-related conditions and 7% trauma or injury.

**Conclusions:**

Great uncertainty remains regarding the return of FDMN to their home country of Myanmar. The current on-going protests following the military coup adds further insecurity to the status of the Rohingya. The presence of a large migrant population relative to a smaller host community burdens the limited facilities and resources of the public health sector. Continued support by the international public health community and civil society organizations is needed.

## Background

The ‘Rohingya’ people are a Muslim ethnic group who reside in Rakhine State, Myanmar, a predominantly Buddhist country. Most Rohingya arrived in Myanmar during the 19^th^ and 20^th^ centuries when the country was a British colony [1,2]. Rohingyas in Myanmar have been persecuted since the 1970s, compelling them to flee their homes. The largest numbers of Rohingyas were forced to migrate in 2017 following a widespread operation by Myanmar’s military that was later declared genocide in a hearing at the International Court of Justice [3]. Myanmar’s armed forces, police and the local Burmese population killed at least 24,000 Rohingya people and perpetrated gang rapes and other forms of sexual violence against 18,000 Rohingya women and girls [4]. At least 392 Rohingya villages were razed to the ground [5].

By January 2018 over a million Rohingyas were forced to escape to neighbouring countries, over half of whom have sought refuge in Bangladesh. They are settled in camps for Forcefully Displaced Myanmar Nationals (FDMN) in Cox’s Bazar. The Ministry of Health and Family Welfare of the Government of Bangladesh and an estimated 150 national and international agencies provide health services in the camps [6]. The FDMNs share the limited local government health care system resources with the local Bangladeshis. A 2017 survey of FDMNs found poor health literacy and insufficient health care while living in Myanmar [7]. A recent review found that the Rohingya refugees are facing deprivation of basic health and other needs. Although various agencies have extended humanitarian assistance, these have been variable in scale and affected by limitations of resources and coordination [8].

We conducted a descriptive study of the FDMNs presenting for care at public health facilities in Bangladesh to get a better understanding of the health problems faced by the FDMNs and the burden on these public health facilities. This assessment could assist national and international health agencies in planning and implementing services for the FDMN.

## Methods

### Definitions

Hypertension was here defined as a systolic blood pressure >140 mmHg combined with a diastolic BP >90 mmHg [9]. Wasting or acute malnutrition in infants and children 6–59 months of age was classified as severe or moderate [10]. Severe acute malnutrition was defined as a mid-upper arm circumference (MUAC) <115 mm, or a weight-for-height/length Z-score below – 3 standard deviations (SD) of the WHO Child Growth Standards median, or with bilateral pitting oedema [11]. Moderate acute malnutrition was defined as a MUAC between 11 cm and 12.5 cm or by a weight-for-height z-score between −3 and −2SD.

### Study site

Bangladesh is divided into 8 Divisions, 64 Districts and 492 sub-districts (*Upazila*) [12]. In the rural areas, the Upazilas are further divided into unions. The primary health care system in each sub-district comprises (1) the community clinic (CC) at the lowest level providing basic health care for a population of around 6000, (2) the union health centre (UHC) and (3) the Upazila (Sub-district) Health Complex (UzHC), which provides in- and out-patient health care.

**Figure 1.**
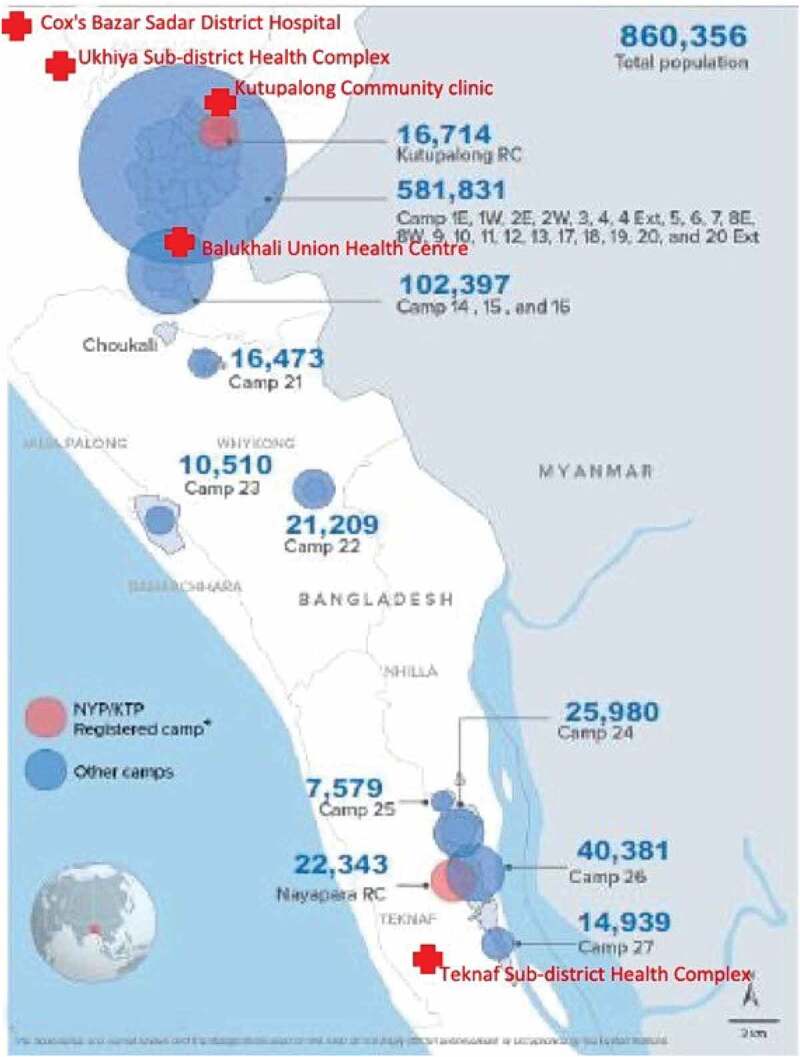
Location of the government health facilities included in the study, in relation to the camps of the Forcefully Displaced Myanmar Nationals [14]

The FDMNs are located at different camps in Cox’s Bazar district (Figure 1). Cox’s Bazar has the world’s largest and most crowded refugee camps, housing about 900,000 Rohingya refugees [13]. The study was conducted at the following government health facilities: (1) Kutupalong CC, (2) Balukhali UHC, (3) Ukhiya UzHC, (4) Teknaf UzHC, and (5) Cox’s Bazar SadarDH, which is a 250-bed teaching hospital of Cox’s Bazar Medical College and is the referral facility for the entire district (Annex D).

### Study procedures

This is a descriptive study with two components. The first is a retrospective review of clinic records from 2015 to 2019 and hospital records from 2017 to 2019. The patients include both local Bangladeshis and FDMNs presenting for care. Retrospective patient data was collected from the hospital registers. The diagnosis of patients during consultation and admission at each health facility is available from the Directorate General of Health Services, Government of Bangladesh [14,15].

The second component comprises a prospective data collection from July 2018 to December 2019 from all FDMN inpatients admitted at Ukhiya UzHC and Teknaf UzHC and Sadar DH and a subset of FDMN outpatients at Kutupalong CC and Balukhali UHC. For the subset, study staff interviewed every 5^th^ FDMN patient attending the facility with a limit of 10 interviews per day. A standard case report form (CRF) in English was developed and translated into Bengali for face-to-face interviews. The CRF included questions regarding demographics, date of consultation, duration of hospital stay, diagnosis and outcome. Illnesses were categorised as communicable diseases; pregnancy-related; nutritional conditions; non-communicable diseases; and trauma or injury (Annex: A). Non-communicable diseases were defined as non-infectious and non-transmissible medical conditions such as cardio vascular diseases, diabetes, chronic kidney and lung disease.

The study staff consisted of 12 trained data collectors: six senior staff nurses in the Sadar DH, two senior staff nurses in each of Ukhiya and Teknaf UzHCs, and two community health care providers in Kutupalong CC and Balukhali UHZ. All data collectors were existing government employees working in the study sites. Their education level was at least 12th grade, they were able to read and translate English and communicate in Rohingya language. A structured training workshop was conducted prior to the study start. The CRF was completed on presentation to the health facility, taking about 10–15 minutes, and for those admitted for inpatient care the outcome until discharge or death was recorded. The field work and data collection were supervised by the local investigators.

### Data management and statistics

The completed paper CRFs were collected on a weekly basis for data cleaning and entry. Data were checked by one investigator (AMSU) before entering into computers in a dedicated area at Cox’s Bazar office, using data entry programs specially created for the project. The data was double entered in ACCESS (Microsoft Corporation Redmond, Washington 98,052–6399, USA).

The primary endpoint was the number of FDMN cases presenting for care at the participating health care facilities, by demographics, date of consultation, and category of illness. The analysis was descriptive. Data were collected, compiled and statistical analysis was done in SPSS V.22 (Statistical Package for Social Science Version 22, Armonk, New York 10,504, USA) for Windows and STATA 15.1 (College Station, Texas 77,845 USA).

## Results

### Retrospective data collection

**Figure 2.**
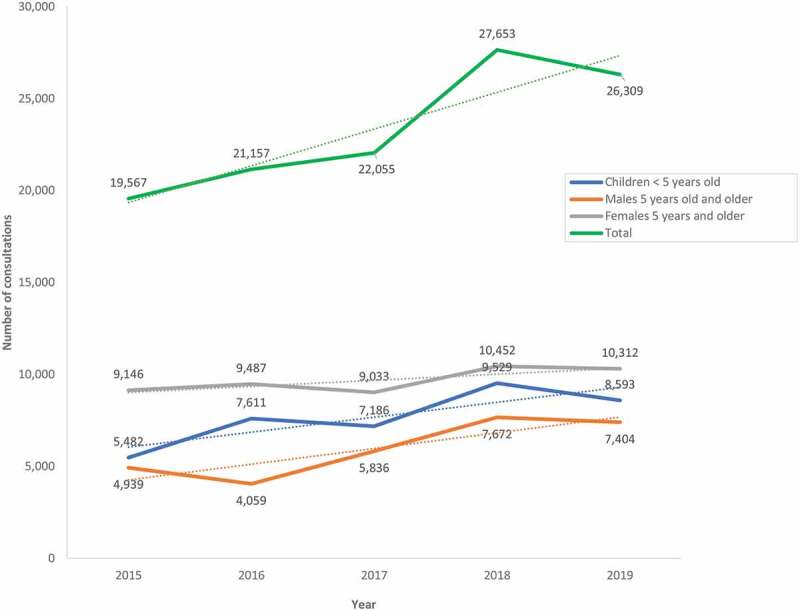
**Number of consultations at the Kutupalong Community Clinic from 2015 to 2019, by sex and age group** (trend lines are represented as interrupted dotted lines)

We plotted the number of out-patient consultations at the at Kutupalong CC, the primary health facility closest to the camps, from 2015 to 2017 (Figure 2). Overall, the number of consultations increased 26% from 19,567 in 2015 to 26,309 in 2019, peaking in 2018 (Figure 1). The largest increase in consultations was for children under five years of age, from 5,482 in 2015 to 8,593 in 2019 (36%).

**Figure 3.**
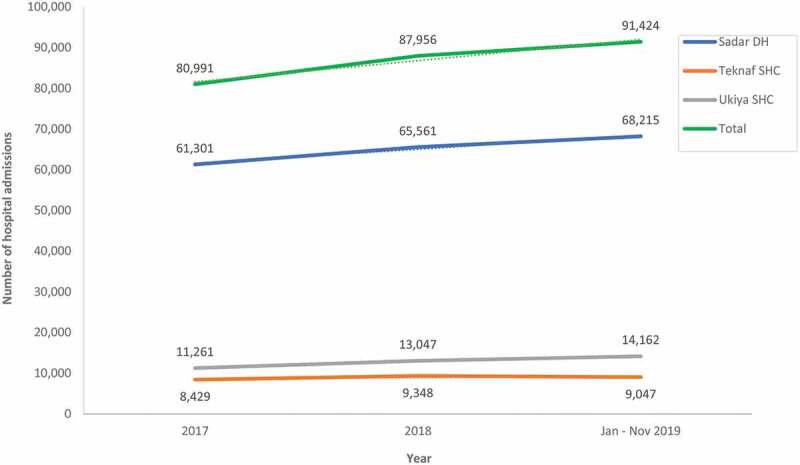
**Number of admissions at Sadar district hospital (DH), TeknafSub-district Health Complex (UzHC), and UkhiyaUzHC from 2017 to 2019** (trend lines are represented as interrupted dotted lines)

There was a corresponding 11% overall increase in admissions at the Sadar DH, Teknaf UzHC, and Ukhiya UzHC, from 80,991 in 2017 to 91,424 in 2019 (Figure 3). Of the total 260,371 admissions from 2017 to 2019, 195,077 (75%) was at the DH, while 26,824 (10%) and 38,470 (15%) were at Teknaf UzHC and Ukiya UzHC, respectively.

### Prospective surveillance

From 1^st^July 2018 to 31^st^December 2019 a total of 9,421 FDMN patients were recruited and interviewed (Table 1). Of the 9,421 patients, 4,075 (43%) were recruited at the Sadar DH, 727 (8%) at Ukhiya UzHC, 498 (5%) at Teknaf UzHC, 1,956 (21%) at Balukhali UHCand 2,165 (23%) at Kutupalong CC.

**Table 1.** Age and sex distribution of FDMN patients interviewed (n = 9421)

	Male		Female		Total	
≥ 5 yo	2,884	(75%)	4,850	(87%)	7,734	(82%)
Children <5yo	966	(25%)	721	(13%)	1,687	(18%)
**Total**	**3,850**	**(100%)**	**5,571**	**(100%)**	**9,421**	**(100%)**

Only 33% (939/2,884) of the male patients 5 years and older had received a formal education and even less, only 24% (1,175/4,850) of the female patients (p < 0.0001). Irrespective of sex the large majority 73% (1,546/2,107) of patients who received any education received a religious education. Before coming to Bangladesh 18% (1,673/9,420) of the FDMN patients worked in farming, 40% (3,760/9,420) had been housewives and 22% (2,040/9420) were children too young to be gainfully employed.

Of the patients 5 years and older 2.5% (200/7,734) were malnourished in contrast to 9% (155/1,687) children under 5 years of age (p < 0.0001). Of the 155 malnourished children 131 (85%) were found to have moderate acute malnutrition and 21 (15%) severe acute malnutrition. Of 5,911 tested patients 72 (1.2%) were found to be hypertensive. The major categories of illness of 9,421 FDMNs who attended for health care between July 2018 and December 2019 were communicable diseases 2,765 (29%), nutritional problems 1,850 (20%), pregnancy-related conditions 1,126 (12%), and trauma or injury 646 (7%) (Table 2).

**Table 2.** Major diagnosis of all Forcibly Displaced Myanmar Nationals (FDMNs) attended for health care (n = 9421) captured during prospective data collection July 2018 – December 2019

	Adults and older children				Young Children					
	Male ≥5yo		Female ≥5yo		Male <5yo		Female <5yo		Total	
Acute Respiratory Infection	388	13%	452	9%	355	44%	303	48%	1498	16%
Pregnancy Complications			1,126	23%					1126	12%
Skin disease	190	6%	393	8%	62	8%	56	9%	701	7%
Injury	357	12%	215	4%	45	6%	29	5%	646	7%
Acute Abdomen	245	8%	335	7%	13	2%	8	1%	601	6%
Peptic ulcer disease	140	5%	283	6%	6	1%	4	1%	433	5%
Back pain	156	5%	234	5%	2	0%	1	0%	393	4%
Viral Fever	113	4%	208	4%	31	4%	35	5%	387	4%
Diarrhoea	73	2%	113	2%	110	13%	74	12%	370	4%
COPD*	166	5%	152	3%					318	3%
Kidney failure	126	4%	94	2%	10	1%	3	0%	233	2%
Pneumonia	44	1%	24	0%	59	7%	34	5%	161	2%
Chronic Lung Disease	81	3%	79	2%					160	2%
Hypertension	49	2%	61	1%	0	0%	1	0%	111	1%
Anaemia	38	1%	58	1%	9	1%	3	0%	108	1%
Diabetes	37	1%	66	1%					103	1%
Kidney Stone	53	2%	43	1%	3	0%	0	0%	99	1%
Asthma	43	1%	45	1%	4	0%	4	1%	96	1%
Heart disease	40	1%	50	1%	2	0%	0	0%	92	1%
Hernia	75	2%	1	0%	10	1%	1	0%	87	1%
Poisoning	47	2%	27	1%	5	1%	7	1%	86	1%
Viral hepatitis	25	1%	48	1%	1	0%	0	0%	74	1%
Stroke	48	2%	21	0%	0	0%	1	0%	70	1%
Cancer	32	1%	34	1%					66	1%
Tuberculosis	38	1%	21	0%	0	0%	2	0%	61	1%
Psychiatric condition	25	1%	29	1%					54	1%
Neuropathy	20	1%	10	0%	1	0%	2	0%	33	0%
Haemorrhoids	15	0%	12	0%					27	0%
Meningitis/Encephalitis	6	0%	6	0%	6	1%	7	1%	25	0%
Epilepsy	4	0%	7	0%	2	0%	1	0%	14	0%
Sepsis	3	0%	6	0%	0	0%	1	0%	10	0%
HIV	4	0%	5	0%		0%		0%	9	0%
Liver abscess	4	0%	3	0%		0%		0%	7	0%
Malaria	3	0%	0	0%		0%		0%	3	0%
Others	331	11%	663	13%	79	10%	60	9%	1133	12%
Total	3019	100%	4924	100%	815	100%	637	100%	9395	100%

*COPD = Chronic Obstructive Airway Diseases

**Figure 4.**
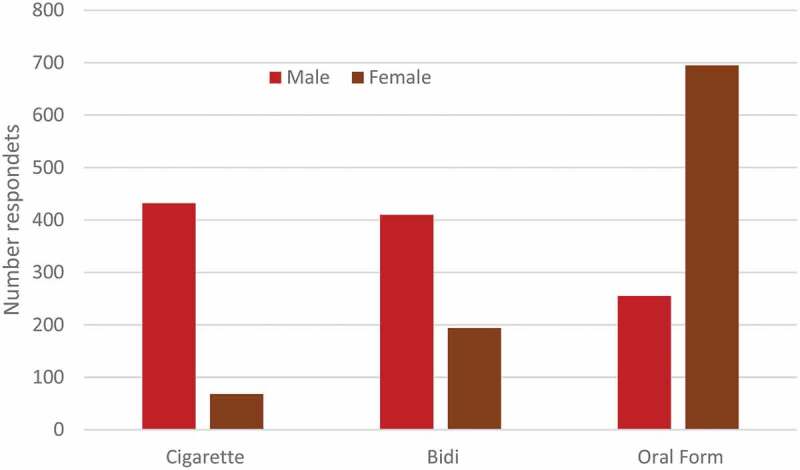
Tobacco consumption among FDMN patients (n = 2,054); bidi are hand rolled cigarretes; oral tobacco includes smokeless tobacco products such as ‘Zarda’ (Tobacco, lime, spices, vegetable dye, areca nut), ‘Gul’ (Tobacco powder, molasses), ‘SadaPata’ (Sun-dried or cured raw tobacco leaf), ‘Khoinee’ (Tobacco, slaked lime, menthol, flavorings, and areca nut)

Of 5,906 adult patients 2,054 (35%) stated that they smoked or consumed tobacco products which was about twice as likely among male respondents 52% (1,097/2,105) compared to female respondents 25% (957/3,801; p < 0.0001). Male respondents were more likely to consume tobacco in the form of cigarettes whereas females were more likely to use oral tobacco products (Figure 4).

**Figure 5.**
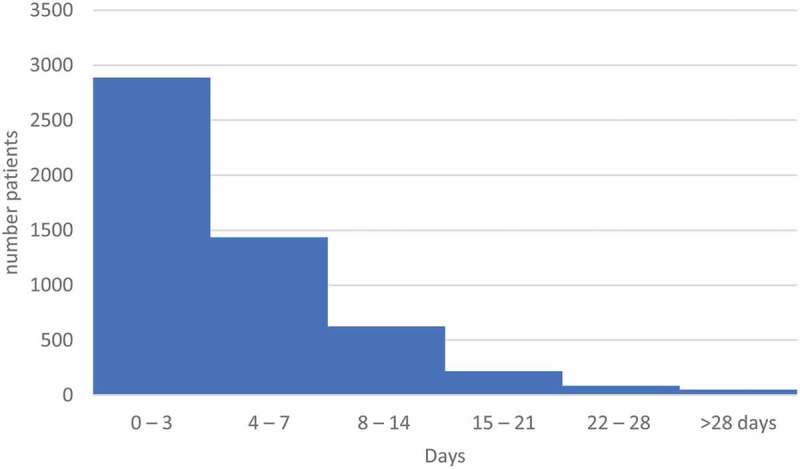
Duration of Hospital Stay of FDMN (n = 5,282)

The median duration of hospital stays was 3 days (IQR 2 to 6 days; Figure 5). The duration of hospital stay among the FDMN was thought to be of longer duration (3 days or longer in 70% of study participants).

104 patients died during the study period including pneumonia (n = 12), COPD (n = 12), injury (n = 6), stroke (n = 6), acute abdomen (n = 6), anaemia (n = 5), chronic lung disease (n = 5), pregnancy complications (n = 4), cancer (n = 4), meningitis or encephalitis (n = 4), kidney failure (n = 4), and heart disease (n = 2). The cause of death was not recorded for 34 patients.

## Discussion

The retrospective review of data confirmed an increase in consultations and admissions following a wave of immigration. The large increases are likely related to the exodus of Rohingyas and arrival in the camps starting in mid-2017. The public health services of Cox’s Bazar, which were overworked and underfunded prior to the refugee crisis, were further stretched. The higher demand of health services was particularly noted for the Kutupalong CC, which is closest to the camps. The admissions to Sadar DH, Teknaf UzHC, and Ukhiya UzHC also increased. From July 2018 to December 2019, we interviewed 9,421 FDMNs seeking health care. We found that 29% had an infectious disease, 20% nutritional problems, 12% pregnancy-related conditions and 7% trauma or injury. The top ten diagnoses were acute respiratory infections, pregnancy complications, skin diseases, injury, acute abdomen, peptic ulcer disease, back pain, viral fever, diarrhoea, and chronic obstructive pulmonary disease. Of those who attended the health facility 12.8% were found to have hypertension which is lower than the 21% prevalence found in a risk factor survey in 2018 in the local Bangladeshi community [16]. The average hospital stay in UzHC and DH is 1–3 days, but the duration of hospital stay among the FDMNs was thought to be longer (3 days or longer in 70% of study participants) probably due to relatively complex medical conditions and being in a more advanced stage compared to local patients. The extended duration of hospital stays was a major burden for the health care system.

In 2017 252,567 OPD attendances, 75,578 emergency attendances and 61,301 admissions were registered at Cox’s Bazar district hospital with bed occupancy ratios of 202% and 149% at Cox’s Bazar Sadar Hospital and Ukhyia UzHC respectively [14]. Cox’s Bazar Sadar Hospital is the referral hospital for the 2.39 million population of 8 subdistricts of Cox’s Bazar. The hospital was set up as a 100-bed facility and recently upgraded to a 250-bed facility but without increases of human resources or logistics. The hospital is used as the teaching hospital of Cox’s Bazar Medical College, a public health training institution. The number of admissions in all these facilities increased significantly after the arrival of FDMNs in Bangladesh. The number of attendances in emergency room of Ukhiya UzHC was 18,920 in 2016 and increased to 30,875 in 2019, which corroborates our findings [15]. One community health care provider in a community clinic provides basic health promotion, preventive services, and limited curative care to a population of ~6000 [17]. Kutupalong CC within close vicinity of the FDMN camp provided services to many FDMN and host population, 26,309 in 2019 alone. The FDMNs were found to have a low level of educational attainment. Most of the FDMNs were involved with agricultural and farming activities (18%) or were children (22%) and housewives (40%) prior to coming to Bangladesh. The low health literacy among FDMNs was described in a recent publication [7]. Viral hepatitis particularly hepatitis C was common among FDMN in comparison with the host community. In a pilot study 3 of 53 (3.8%) people were found to be infected with hepatitis C much higher than local community [18]. Among 200 tuberculosis cases among FDMNs diagnosed by GeneXpert screening for hepatitis found 21% hepatitis C, 11% hepatitis B and 3% hepatitis C + B, and one case of HIV [19]. Injuries were found to be common ailments among FDMNs on arrival in Bangladesh [20]. Injuries, stroke and various musculoskeletal pain are potential causes of disability and deserve further assessment [21].

This study was conducted in emergency conditions and has several limitations. Consultations increased significantly after 2017 although the available data from the Yearbook of DGHS, GOB do not segregate the services provided to the host community and the FDMNs. The clinic and hospital records also did not record this information. Without this information it is not possible to conduct analytical tests and associations cannot be confirmed. Study participants were not identified by a unique ID number. It is possible that individual patients made use of the health care system repeatedly. The study reports therefore disease episodes and not individual patients.

## Conclusion

Great uncertainty remains regarding the return of FDMN to their home country of Myanmar. The military coup which started on 1 February 2021 and led to widespread protests adds further insecurity to the status of the Rohingya. The difficult situation of FDMNs has been further complicated with the COVID pandemic which has added unprecedented demand on health care services including the services of the host country Bangladesh. In the absence of increasing resources, it is challenging for the health care services to provide high quality care. We believe there is an acute requirement to assess the health needs of the FDMN in Bangladesh. The presence of a large migrant population relative to a smaller host community burdens the limited facilities and resources of the public health sector which no longer can fully address the requirements of the local Bangladeshi population. The health needs of the local populations needs also to be urgently assessed. There is no tradition of buying health insurance in Bangladesh, people are accustomed of purchasing services. This is specifically true for investigations not available in public hospitals and require cash payment or borrowing of money not available to FDMN. Continued support by the international public health community and civil society organizations is critically needed.

## Supplementary Material

Supplemental Material

## Paper context

The forced displacement of Myanmar nationals into Bangladesh in 2017 was expected to have major consequences for the local health care services. Using retrospective and prospectively collected data we estimate the increase in the use of government health care services following the arrival of the refugees. There was a 26% increase in consultations in the community clinic closest to the camps. The most frequent presentations included infectious diseases, nutritional problems, pregnancy-related conditions, and trauma.
