# Supplementary material for: A descriptive study of Forcefully Displaced Myanmar Nationals (FDMN) presenting for care at public health sector hospitals in Bangladesh
Source: Glob Health Action. 2021 Sep 8;14(1):1968124. doi: 10.1080/16549716.2021.1968124 (PMC8439211; doi:10.1080/16549716.2021.1968124)

**Annex A**

**Patient name:**....................................................., **Study code**: **[ ] [ ] [ ] - [ ] [ ] [ ]**

**A descriptive study of forcefully displaced Myanmar Nationals presenting for care at public health sector hospitals in Bangladesh**

**CASE REPORT FORM**

**Dist.Sadar Hospital Cox’s Bazar |__|; UHC Ukhiya|__|; UHC Teknaf|__|; CC |__|; Union HC |__|**

Verbal Consent taken from Patient (if more than 18 Years) or from guardian: *Yes = 1, No = 2* I___|

Ward: Medicine |__**|;**Gynae&Obst|__**|;** Surgery |__**|;** Pediatrics |__**|**; Others **|__|**

Serial number: |__I__||__I

Date of admission: |__I__|/|__I__|/|__I__| Date of Discharge: |__I__|/|__I__|/|__I__|

Date of referral: |__I__|/|__I__|/|__I__| Date of Death: |__I__|/|__I__|/|__I__|

1. Duration of hospital stay (days ) : |__I__|
2. Admission/Attendance by : (*Referral = 1, Came by own = 2*) I___|
3. Age (yrs or months) :I___|I___| .. I___|I___|
4. Sex: *Male = 1, Female = 2* I___|

Name:...................................................................................................., Mobile no.: ...........................

ID number (if any):.................................................................................

1. Number of family members (when in Myanmar): I___|I___|
2. Occupation (when in Myanmar): (See Occupational Code) I___|I___|
3. Own and Use LLN (when in Myanmar): *Yes = 1, No = 2* I___|
4. Any formal education: *Yes = 1, No = 2*I__| (*If yes: Religiousedu. =1 or Else=2*): I___|
5. Nutritional status: *Normal = 1, Malnutrition = 2, Obese = 3* I___|
6. Degree of malnutrition (if <5 yrs) (*Mild =1, MAM = 2, SAM = 3*) I___|
7. Tobacco consumption :*Yes = 1, No = 2*|__I (If Yes *Cigarette=1,Bidi=2, Oral Form =3*) I___|
8. Systolic blood pressure(mm Hg) If age >20: |__I__||__I
9. Diastolic blood pressure (mm Hg) If age >20: |__I__||__I
10. Known case of hypertension(High blood pressure) If age >20: *Yes = 1, No = 2* I___|
11. Known case of Diabetes Mellitus If age >20 :*Yes = 1, No = 2* I___|
12. Weight (Kg) :I___|I___|
13. Clinically anaemic: *Yes = 1, No = 2* I___|
14. Findings of Chest X-Ray (if done) :*Normal= 1, Abnormal= 2* I___|
    - 1. (Description if abnormal ………………………………………………)
15. Confirmatory investigation for diagnosis (if any):....................
16. Status of Hepatitis B/C known before: *Yes = 1, No = 2* I___|
17. Diagnosis: (ICD 10 If possible). (See the Disease Code) .................................................................................................................................................................. ..................................................................................................................................................................
18. Outcome (Admitted case- Upazilla HC/Dist. Hospital): *Improved =1, Referred =2, Died=3* I___|

Outcome: OPD case (CC, Union HC) *Treated=1, Referred = 2* I___|

1. Cause of Death (Not Cardio Respiratory Failure only):...........………………………...

*Data Recorded by Name &Mobile:………………………...…, Sig.: ………………, Date:..........*

| **Occupations Codebook** | |
| --- | --- |
| **Profession/main source of income** | **Code** |
| Farmer: Paddy/Rice, Vegetables | 01 |
| Farmer: Farm- Duck-Hen | 02 |
| Farmer: Farm- Cow-Buffalo, Goat | 03 |
| Forest Worker | 04 |
| Small business owner | 05 |
| Teacher, Religious Teacher, Office Worker, Other white-collar job | 06 |
| Health Worker | 07 |
| Child | 08 |
| Fisherman | 09 |
| Student | 10 |
| Housewife | 11 |
| Retired | 12 |
| Jobless | 13 |
| Rickshaw/Van/Auto driver | 14 |
| Riverboat labour | 15 |
| Farm labour | 16 |
| Day labour | 17 |
| Carpenter/Other Artisan | 18 |
| Other – (please specify in the space provided)................................... | 19 |

| **Disease Code** | | | | |
| --- | --- | --- | --- | --- |
| **Disease Name** | **Code** |  | **Disease Name** | **Code** |
| Injury | 1 |  | Diabetes | 19 |
| Pneumonia | 2 |  | Hypertension | 20 |
| Hernia | 3 |  | Heart disease | 21 |
| Stone | 4 |  | Acute Abdomen | 22 |
| Asthma | 5 |  | Diarrhea | 23 |
| CLD | 6 |  | Peptic ulcer | 24 |
| TB | 7 |  | Meningitis/Encephalitis | 25 |
| COPD | 8 |  | Psychiatric conditions | 26 |
| Cancer | 9 |  | Epilepsy | 27 |
| Stroke | 10 |  | Skin disease | 28 |
| HIV | 11 |  | Back pain/LBP/MSK | 29 |
| Anemia | 12 |  | Sepsis | 30 |
| Neuropathy | 13 |  | Malaria | 31 |
| Viral Fever | 14 |  | Liver abscess | 32 |
| Pregnancy Complications | 15 |  | Hemorrhoids | 33 |
| Viral hepatitis | 16 |  | ARI | 34 |
| Poisoning | 17 |  | Malnutrition | 35 |
| Kidney failure | 18 |  | Others | 36 |

#



**

**

**Annex B**

**

**

**
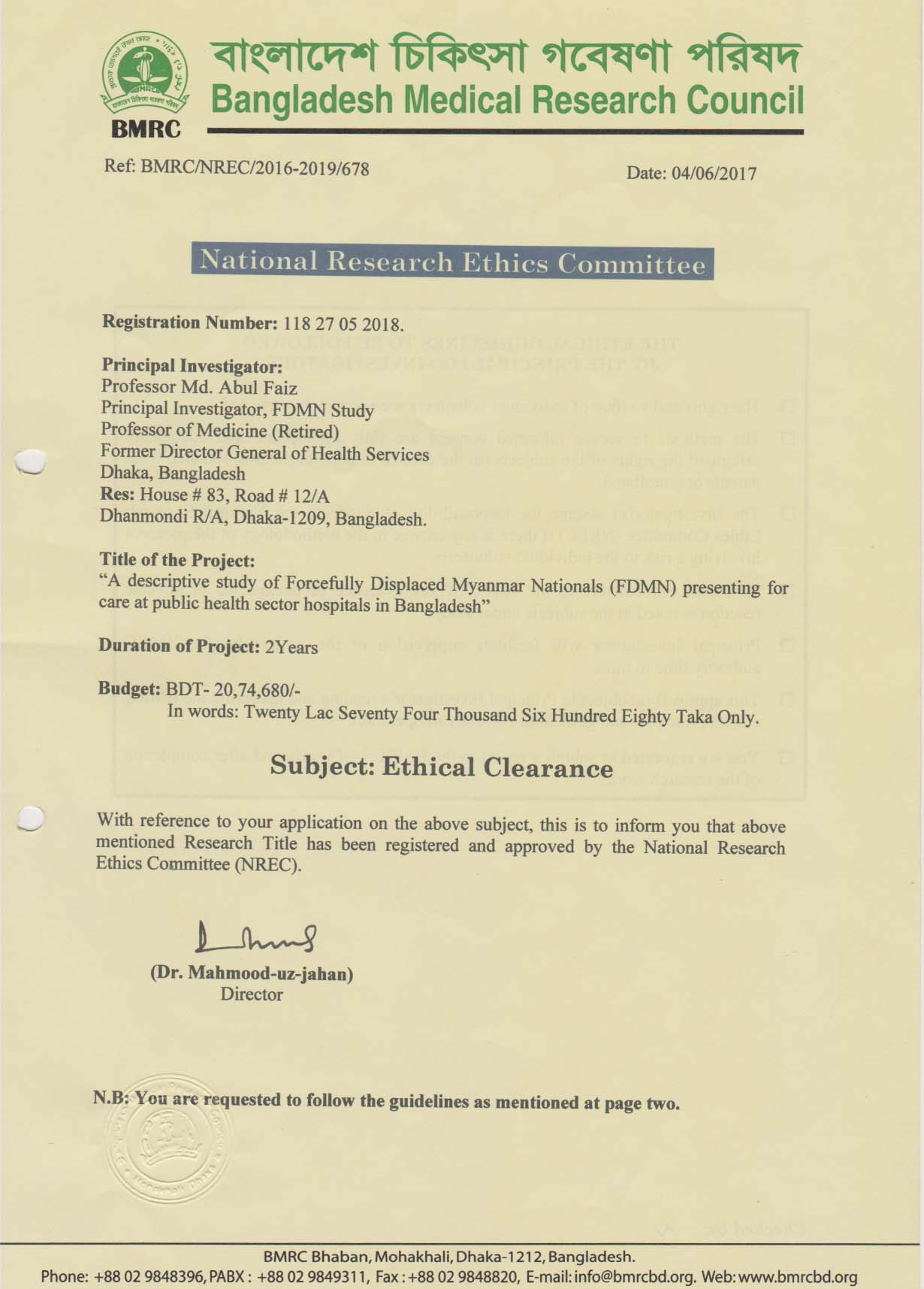
Annex C**

**A descriptive study of forcefully displaced Myanmar Nationals presenting for care at public health sector hospitals in Bangladesh**

**Informed Verbal Consent Form**

Name of the Principal Investigator: **Professor Mohammad Abul Faiz**

Finance: Hanako Foundation, Singapore.

Title: A descriptive study of Forcefully Displaced Myanmar Nationals (FDMN) presenting for care at public health sector hospitals in Bangladesh

**Information and Consent/Assent**

**Introduction:**

I am ….................................................................., a health professional working in this health centre/hospital.

We are going to collect some information about health and illness of FDMNs at Bangladesh while they attend public health facilities for treatment as a part of study.

**Objective of the study:**

In this study we shall collect information about health and illness of patients who attend government health centre or hospital. This will give us information about the pattern of disease the FDMNs are suffering from.

**Nature of study:**

In this study we shall ask some questions related to your/your ward’s health and do some simple physical examination.

**Selection of the participant:**

Those FDMN patients who are admitted and a sample of patients attending OPD of CC/Union HC.

**Voluntary participation/Right to refuse or withdraw:**

You can choose to participate or not. This is your decision, and there is no consequences if you choose not to participate. You can decide not to participate or withdrawal of consent. There will be no negative consequences.

**Procedure and protocol:**

1. We shall ask you some questions related to your/your ward’s health and illness
2. We need to do some physical examination
3. Our total procedure will not take more then 10 minutes.

**Risks:**

There is no risk at all to any of our questions or physical examination.

**Benefits:**

If you participate in this research there is no benefit for you or your child, but your participation is likely to help us in finding the answer to the research question.

**Reimbursement:**

There will be no payment for your participation in this study.

**Confidentiality:**

We will not be sharing the identity of those who participated in this study. The collected information will remain confidential and nobody but the researchers will see it. All of the information collected will have a code, instead of your name and code and names will be locked in a cabinet. Confidential information will not be shared.

**Who to contact:**

If you have any questions you may ask them now or later even after the study has started. I am available in this health centre/hospital.

**Certificate of consent:**

The information was read and explained to me.

1. I know that I will be asked some questions related to my/my ward’s health and illness.

2. I/my ward need to undergo some physical examination

3. I have had the opportunity to ask about the study and my questions about the study and my questions have been answered to my satisfaction.

4. I consent voluntarily to participate/my ward to participate in this study.

**Name of the participant:**.....................................................................**, Phone:**..........................

**ID number (if any):** ......................................................................................

Name of the person taking consent:..........................................................

Signature of the person taking consent: ....................................................

Date (*Day/Month/Year*):..................................





**

**

**Annex D: Health Centres and Hospitals of District Cox’s Bazar:**


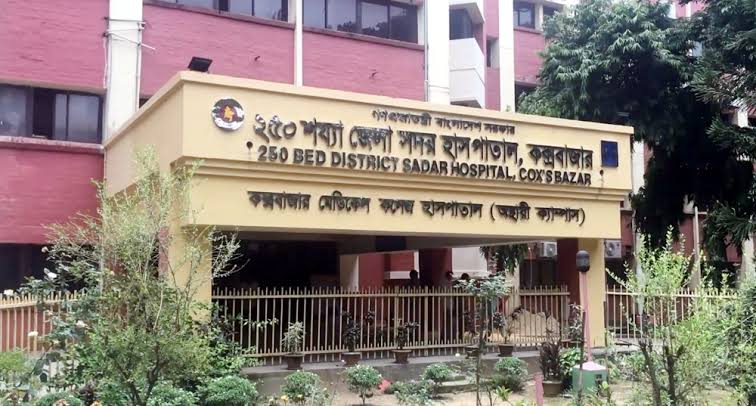

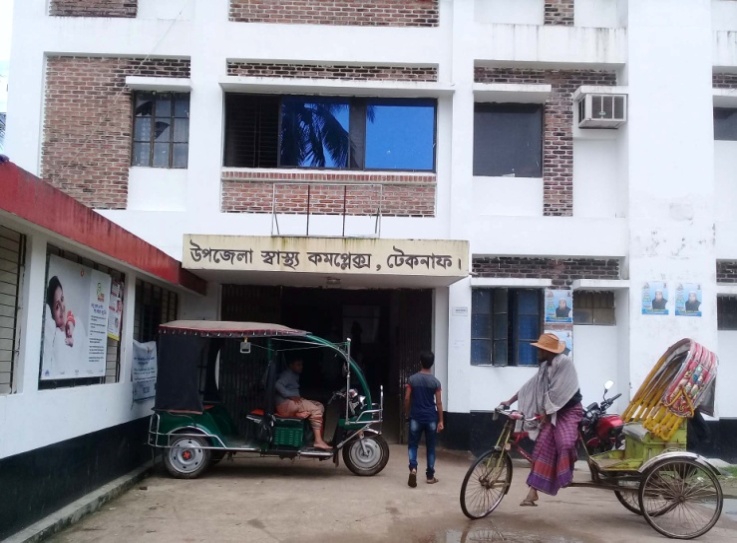


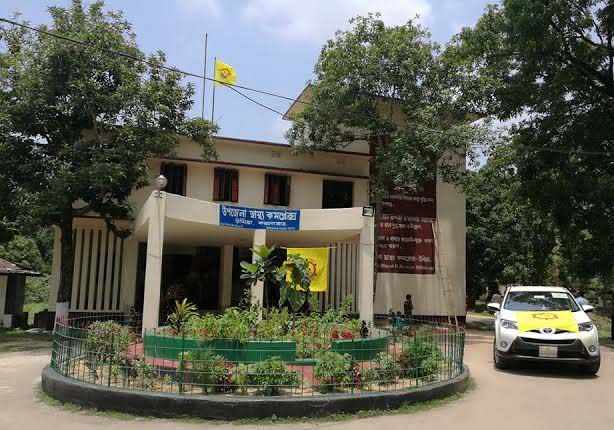


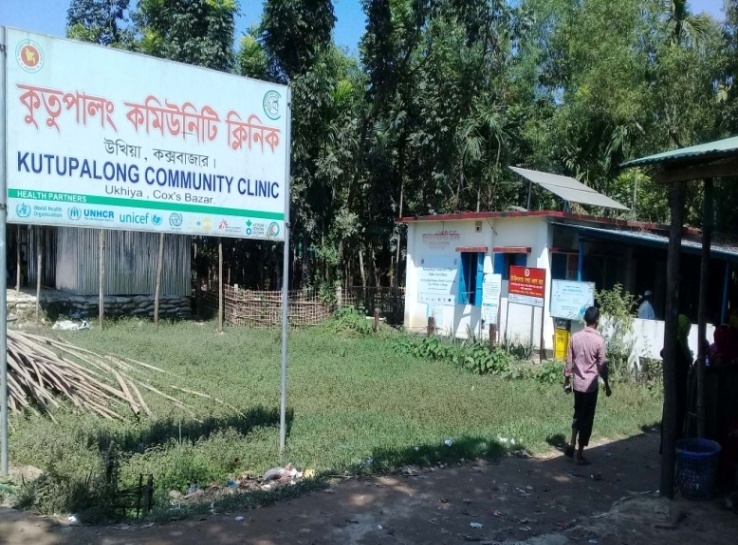

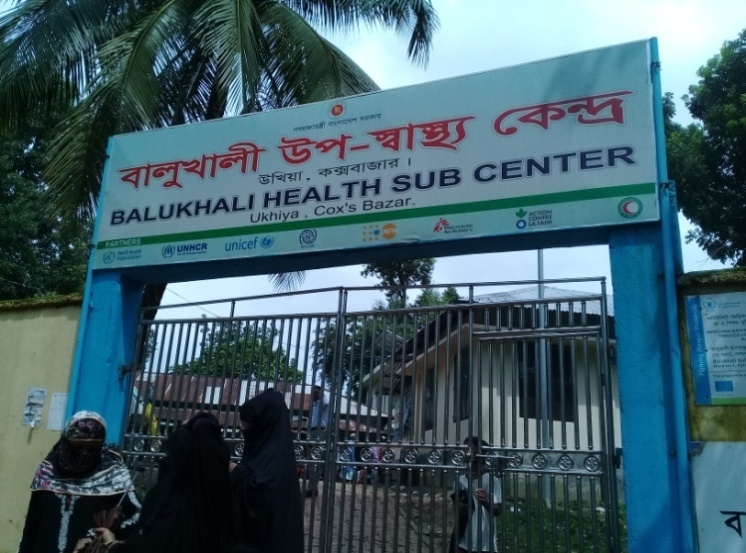

Supplement: Supplemental Material [file ZGHA_A_1968124_SM8771.docx]
